# Seroprevalence and Risk Factors Associated with Anti-*Toxocara* spp. IgG Seropositivity Among Cat Owners in an Island Community: A Cross-Sectional Study in Koh Yao District, Phang Nga Province, Thailand

**DOI:** 10.3390/ijerph23081007

**Published:** 2026-07-31

**Authors:** Prasit Na-Ek, Chuchard Punsawad, Aulia Rahmi Pawestri, Udomsak Narkkul

**Affiliations:** 1 Department of Medical Science, School of Medicine, Walailak University, Nakhon Si Thammarat 80160, Thailand; prasit.na@wu.ac.th (P.N.-E.); chuchard.pu@wu.ac.th (C.P.); 2 Center of Excellence in Tropical Pathobiology, Walailak University, Nakhon Si Thammarat 80160, Thailand; 3 Department of Parasitology, Faculty of Medicine, Universitas Brawijaya, Malang 65145, Indonesia; aulia_rp@ub.ac.id

**Keywords:** seroprevalence, *Toxocara* spp., cat owner, risk factors, Thailand

## Abstract

**Highlights:**

**Public health relevance—How does this work relate to a public health issue?**

- *Toxocara* spp. are neglected zoonotic helminths that can infect humans through contact with contaminated environments and companion animals.
- Epidemiological data on *Toxocara* spp. seropositivity among cat owners in remote island communities of Thailand remain limited despite frequent human–cat interactions.

**Public health significance—Why is this work of significance to public health?**

- A high seroprevalence (34.36%) of *Toxocara* spp. seropositivity was identified among cat owners in Koh Yao District, southern Thailand.
- Handwashing after cat contact was independently associated with a reduced risk of *Toxocara* spp. seropositivity.

**Public health implications—What are the key implications or messages for practitioners, policy makers and/or researchers in public health?**

- Health education programs promoting hand hygiene after cat contact should be strengthened in communities with high exposure to companion animals.
- Further environmental investigations and longitudinal studies are needed to better understand transmission pathways and support evidence-based control strategies within a One Health framework.

**Abstract:**

*Toxocara* spp. infection is a neglected zoonotic disease associated with exposure to companion animals and contaminated environments. However, information regarding its seroprevalence and associated risk factors among cat owners in Thailand, particularly in remote island communities, remains limited. This cross-sectional study aimed to determine anti-*Toxocara* spp. IgG seropositivity and identify associated risk factors among cat owners in Koh Yao District, Phang Nga Province, Thailand. A total of 291 cat owners participated in the study. Sociodemographic characteristics, health-related behaviors, serum anti-*Toxocara* spp. IgG antibodies, and hematological parameters were collected. Univariate and multivariable logistic regression analyses were performed to identify factors associated with seropositivity. The overall prevalence of anti-*Toxocara* spp. IgG seropositivity was 34.36%. In the univariate analysis, failure to wash hands after cat contact was significantly associated with higher odds of seropositivity (odds ratio [OR] = 1.96, 95% confidence interval [CI]: 1.16–3.33; *p* = 0.011). This association remained significant in the multivariable logistic regression model. These findings indicate substantial exposure to *Toxocara* spp. among cat owners in this island community and highlight the importance of personal hygiene, particularly handwashing after cat contact, in reducing exposure to infective *Toxocara* eggs. Targeted health education programs and preventive interventions should be strengthened to reduce the risk of *Toxocara* spp. exposure in endemic communities.

## 1. Introduction

*Toxocara* spp. are parasitic nematodes that cause the zoonotic disease toxocariasis in humans. The disease is most prevalent in tropical and subtropical regions, particularly in low- and middle-income countries [1,2]. *Toxocara canis* and *T. cati* have definitive hosts in domestic dogs and domestic cats, respectively [3]. A recent study estimated the global prevalence of *Toxocara* infection in dogs to be 11.1% [4]. Several studies have also investigated *Toxocara* infection in cats, reporting prevalence rates of 23.0% in Baghdad, Iraq [5], 17.7% in Isfahan, Iran [6], and 17.7% in Manila, Philippines [7]. A global meta-analysis estimated the overall prevalence of *Toxocara* infection in cats to be 17.0% [8]. Human exposure primarily occurs through the ingestion of embryonated *Toxocara* eggs contaminating soil via dog or cat feces or through the consumption of raw or undercooked meat containing *Toxocara* larvae in paratenic hosts [9]. It has been estimated that toxocariasis affects approximately 19.0% of the global population [10].

Although most individuals infected with *Toxocara* spp. remain asymptomatic, several clinical manifestations of human toxocariasis have been described, including visceral larva migrans (VLM), ocular larva migrans (OLM), neurotoxocariasis, and covert (common) toxocariasis [2,11]. In addition, *Toxocara* infection has been implicated in the development of allergic disorders, particularly asthma and other atopic conditions [11]. Because *Toxocara* larvae cannot complete their life cycle in humans, infected individuals are considered paratenic (dead-end) hosts and do not shed *Toxocara* eggs in their feces [2,11]. Consequently, direct parasitological diagnosis is rarely feasible. Therefore, serological methods, particularly enzyme-linked immunosorbent assays (ELISAs) based on *Toxocara* excretory–secretory (TES) antigens, are widely used for the diagnosis and epidemiological investigation of human toxocariasis [11,12]. However, TES-based immunodiagnostic assays have limitations, including cross-reactivity with antibodies against other helminths, especially *Ascaris lumbricoides*, and the inability to distinguish between past and current infections.

Numerous studies have reported the seroprevalence of anti-*Toxocara* spp. IgG seropositivity in human populations worldwide. Particularly high seroprevalence rates have been reported in rural areas of Rio Grande do Sul, Brazil (71.8%) [13], Makoko, Nigeria (86.1%) [14], and the Republic of the Marshall Islands (86.75%) [15]. In contrast, lower seroprevalence rates have been observed in several countries, including the United States (5.1%) [16], Mexico (4.7% in adults and 13.8% in children) [17], Italy (8.0%) [18], and Greece (16.0%) [19]. Relatively high seroprevalence has also been reported in Southeast Asia, including Nakhon Si Thammarat, Thailand (58.2%) [20], Ho Chi Minh City, Vietnam (45.2%) [21], and Los Baños, Laguna, Philippines (49.0%) [22].

Dogs and cats are among the most popular companion animals worldwide and provide enhanced security, improved mental and physical well-being, and social support [23]. *Toxocara* spp. infection in dogs and cats has been widely reported worldwide, including in Australia (Western Pacific), Thailand (Southeast Asia), and Iran (Eastern Mediterranean region) [4,24,25,26,27]. Cat owners may be exposed to *Toxocara* spp. through contact with contaminated soil or other environmental sources contaminated with embryonated *Toxocara* eggs. Furthermore, close contact with pets and inadequate hand hygiene have been identified as potential risk factors for human toxocariasis [10,20]. Cat owners represent a population with potentially increased exposure to *Toxocara* spp. because of their close and frequent contact with cats and cat-associated environments. Although previous studies have identified pet ownership and animal contact as potential risk factors for human toxocariasis, epidemiological data among cat owners remain limited, particularly in Southeast Asia [10,28,29,30]. Most available studies have focused on schoolchildren, dog owners, or other populations rather than cat owners [20,31,32], leaving an important knowledge gap regarding the burden of *Toxocara* spp. exposure among cat owners and the factors influencing their risk of exposure.

Koh Yao District, Phang Nga Province, is a rural island community in southern Thailand where the majority of residents rely primarily on agriculture, fisheries, and small-scale local occupations [33,34]. Cats are commonly kept as companion animals in households throughout the district, resulting in frequent human–cat interactions within homes and communities. The geographical isolation of island communities may influence access to healthcare and veterinary services, while local environmental and cultural conditions may affect zoonotic disease transmission [33,34]. Moreover, previous environmental investigations conducted in Koh Yao District detected *Toxocara* spp. eggs in school soils, indicating the presence of environmental reservoirs and potential opportunities for human exposure [35]. However, no study has investigated anti-*Toxocara* spp. IgG seropositivity among cat owners in this island setting. Given the presence of environmental reservoirs and the close interactions among humans, animals, and the environment, understanding *Toxocara* spp. exposure in such communities is important for developing targeted prevention and control strategies within a One Health framework [2,10]. Therefore, this study aimed to determine the seroprevalence of anti-*Toxocara* spp. IgG antibodies, identify associated risk factors, and evaluate hematological characteristics among cat owners in Koh Yao District, Phang Nga Province, Thailand.

## 2. Materials and Methods

### 2.1. Study Design, Setting and Population

**Figure 1:**
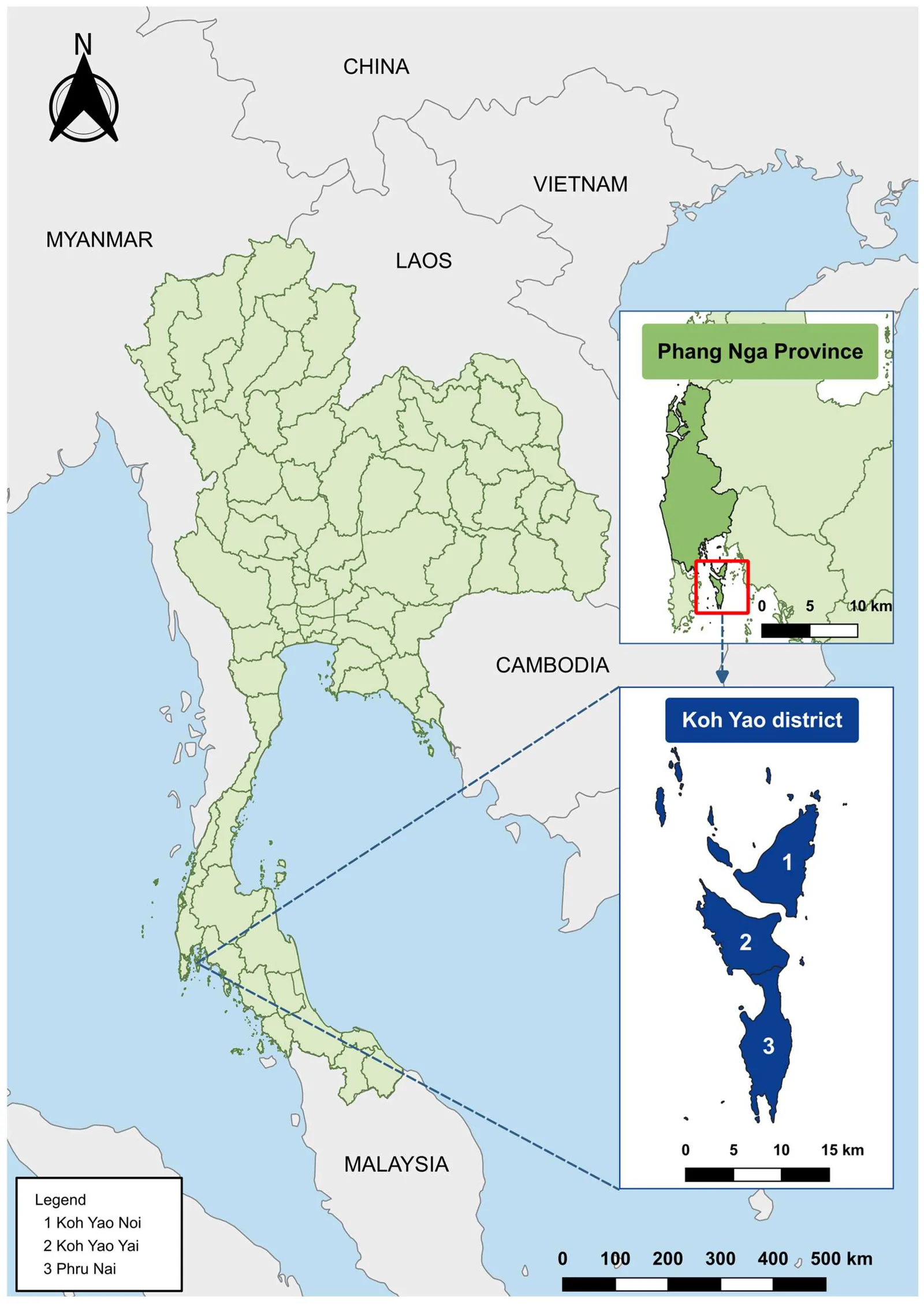
Map of the study area showing the three subdistricts of Koh Yao District, Phang Nga Province, southern Thailand: Koh Yao Noi (1), Koh Yao Yai (2), and Phru Nai (3). The map was created using Quantum GIS version 3.16.11 with ESRI basemaps.

This cross-sectional study was conducted among cat owners residing in Koh Yao District, Phang Nga Province, southern Thailand. According to official population registry data obtained on 18 August 2022, Koh Yao District had 12,325 residents aged 7–80 years. Of these residents, 6248 were male, and 6077 were female. Koh Yao District consists of three subdistricts: Koh Yao Noi, Koh Yao Yai, and Phru Nai (Figure 1).

### 2.2. Study Sample

Eligible participants were male and female cat owners aged 18 years and older residing in Koh Yao District, Phang Nga Province. Exclusion criteria included immunocompromising conditions (e.g., human immunodeficiency virus (HIV) infection, acquired immunodeficiency syndrome [AIDS], or autoimmune disorders), the use of systemic corticosteroids or other immunosuppressive medications for at least 3 months before enrollment, and acute illnesses (e.g., fever or acute infection) on the day of blood collection. Data collection was conducted between April and May 2024.

### 2.3. Sample Size and Sampling Method

The sample size was calculated using the single population proportion formula: Z^2^p(1 − p)/d^2,^ where p represents the estimated seroprevalence of *Toxocara* spp. infection from a previous study, d is the margin of error, and Z is the standard normal deviate corresponding to a 95% confidence level (1.96). The calculation was based on a reported seroprevalence of 76.5% (*p* = 0.765) from a previous study conducted among dog owners in southern Thailand [31]. As no published data on anti-*Toxocara* spp. IgG seroprevalence among cat owners in Thailand were available at the time of study design, this study was considered the most geographically relevant reference for estimating the required sample size. A 95% confidence level (Z = 1.96) and a margin of error of 5% (d = 0.05) were used, resulting in a minimum required sample size of 277 participants. To compensate for potential non-response or incomplete data, an additional 10% was added, resulting in a target sample size of 305 participants. Participants were recruited using a convenience sampling approach from the three subdistricts of Koh Yao District (Koh Yao Noi, Koh Yao Yai, and Phru Nai). Eligible cat owners were identified with the assistance of village health volunteers (VHVs). Only one eligible cat owner was recruited from each household after providing written informed consent. The final number of participants depended on the availability and willingness of eligible cat owners during the study period.

### 2.4. Data Collection on Risk Behaviors for Toxocara spp. Seropositivity

A structured questionnaire was used to collect information on demographic characteristics, health-related behaviors, and potential risk factors associated with anti-*Toxocara* spp. IgG seropositivity. The questionnaire was developed based on a review of the relevant literature and was subsequently evaluated for validity and reliability. Content validity was assessed by three experts in parasitology and related fields to ensure the relevance, clarity, and appropriateness of the questionnaire items. Following revision, the questionnaire was pilot tested among 20 individuals with characteristics similar to those of the study population. The questionnaire demonstrated good internal consistency (Cronbach’s alpha = 0.81), indicating acceptable reliability. The questionnaire consisted of two sections. Part 1 collected general information about the participants, whereas Part 2 focused on health-related behaviors and potential risk factors for *Toxocara* spp. infection. Occupations were categorized into two groups: unskilled and skilled workers. Unskilled workers were defined as individuals without specialized training or formal professional qualifications, including agricultural workers, farmers, traders, and homemakers. Skilled workers were defined as individuals with specialized training, technical expertise, or formal education, including government officers, company employees, and teachers.

### 2.5. Blood Sample Collection and Serum Preparation

Five milliliters of venous blood were collected from each participant. Of the total volume, 3 mL was transferred into a clot activator tube for the detection of anti-*Toxocara* spp. IgG antibodies. Blood samples were stored in a cooling box at approximately 4 °C and transported to the laboratory on the same day for processing. After clot formation, the samples were centrifuged at 2500 rpm for 10 min to separate the serum. The serum was carefully aliquoted into sterile microcentrifuge tubes and stored at −80 °C until further analysis. The remaining 2 mL of blood was transferred into an EDTA tube, maintained at 4 °C, and transported immediately to the laboratory for hematological analysis.

### 2.6. Investigation of Hematological Parameters

Hematological parameters, including red blood cell count (RBC), hemoglobin concentration, hematocrit, mean corpuscular volume (MCV), mean corpuscular hemoglobin (MCH), mean corpuscular hemoglobin concentration (MCHC), platelet count (PLT), red blood cell distribution width-standard deviation (RDW-SD), red blood cell distribution width-coefficient of variation (RDW-CV), platelet distribution width (PDW), mean platelet volume (MPV), platelet large cell ratio (P-LCR), white blood cell count (WBC), neutrophils, lymphocytes, monocytes, eosinophils, and basophils, were measured using an automated hematology analyzer (Sysmex XN-330, Sysmex Corporation, Kobe, Japan) at the Medical Technology Clinic, Walailak University, Thailand [31].

### 2.7. Detection of IgG Antibodies Against Toxocara spp.

Commercial ELISA kits (*Toxocara* IgG ELISA Kit, Abnova Corporation, Taipei, Taiwan), with a reported sensitivity and specificity of >95% according to the manufacturer, were used to detect serum anti-*Toxocara* spp. IgG antibodies. Serum samples were diluted 1:100 with the supplied sample diluent, and all assays were performed in duplicate. Following incubation, washing, and substrate reaction steps, the optical density (OD) was measured at 450 nm against a reagent blank within 30 min using a microplate reader. The Index of Positivity (IP) was calculated as the ratio of the sample OD to the mean OD of the cut-off control. Samples with an IP > 1.1 were considered positive, whereas those with an IP < 0.9 were considered negative. Samples with IP values between 0.9 and 1.1 were considered equivocal and were retested using a follow-up serum sample collected 2–6 weeks after the initial sampling.

### 2.8. Ethical Considerations

This study was approved by the Human Research Ethics Committee of Walailak University, Thailand (approval number WUEC-24-147-01). Written informed consent was obtained from all participants prior to enrollment. Participant confidentiality was maintained throughout the study, and all data were anonymized before analysis.

### 2.9. Statistical Analyses

All data were entered, cleaned, and analyzed using IBM SPSS Statistics for Windows, version 20.0 (IBM Corp., Armonk, NY, USA). Quantitative variables were summarized as mean ± standard deviation (SD), whereas categorical variables were presented as frequencies and percentages. Associations between potential risk factors and *Toxocara* spp. seropositivity were initially assessed using univariate logistic regression analyses. The variables in the univariate logistic regression model with *p*-values less than 0.2 were included in a multiple logistic regression model that was adjusted for confounding factors. Differences were considered statistically significant when the *p*-value was less than 0.05. Hematological parameters were compared using the Mann–Whitney U test and independent *t*-test for nonparametric and parametric data analysis, respectively.

## 3. Results

### 3.1. Sociodemographic Characteristics of the Study Population

Of the 305 individuals approached, 291 completed the study, yielding a response rate of 95.4%. More than half resided in the Phru Nai subdistrict (54.30%), followed by Koh Yao Noi (33.33%) and Koh Yao Yai (12.37%). Females accounted for 85.91% of the study population, whereas males represented 14.09%. The largest age group was 36–59 years (53.26%), followed by those aged ≥60 years (25.77%) and 18–35 years (20.96%). Regarding educational attainment, 52.92% had completed elementary school or less, while 47.08% had attained education beyond the elementary level. Most participants were Muslim (98.28%), whereas 1.72% were Buddhist. The majority were married (74.57%), followed by single (20.27%) and widowed or divorced individuals (5.15%). With respect to occupation, 91.75% were classified as unskilled workers and 8.25% as skilled workers. Regarding monthly income, 40.89% reported earning less than USD 300 per month, whereas 59.11% reported an income of USD 300 or more. Detailed sociodemographic characteristics of the study population are presented in Table 1.

### 3.2. Seroprevalence of Anti-Toxocara spp. IgG Antibodies

Among the 291 cat owners included in the study, 100 participants tested positive for anti-*Toxocara* spp. IgG antibodies, yielding an overall seroprevalence of 34.36%. The seroprevalence varied across the three subdistricts, with the highest prevalence observed in Koh Yao Noi (44.33%), followed by Koh Yao Yai (36.11%) and Phru Nai (27.85%). The seroprevalence was slightly higher among males (36.59%) than females (34.00%). By age group, seropositivity was comparable across categories, ranging from 32.79% among participants aged 18–35 years to 34.84% among those aged 36–59 years and 34.67% among those aged 60 years and older. Participants with an education level of elementary school or below exhibited a higher seroprevalence (38.96%) than those with education beyond the elementary level (29.20%). According to marital status, the highest seroprevalence was found among single participants (37.29%), followed by married (34.10%) and widowed/divorced participants (26.67%). Regarding occupation, unskilled workers demonstrated a higher seroprevalence (35.21%) than skilled workers (25.00%). Participants with a monthly income of less than USD 300 had a slightly higher seroprevalence (36.13%) compared with those earning USD 300 or more (33.14%) (Table 2).

### 3.3. Health Behaviors Related to Toxocara spp. Exposure

Table 3 presents the distribution of health-related behaviors among the study participants. Few participants reported deworming their cats every three months (4.47%), providing flea/tick control (7.56%), or bathing/grooming their cats weekly (9.97%). Interactions between participants and their cats were frequently reported, including playing with cats (40.21%), touching cats (37.46%), kissing cats (23.37%), and sleeping with cats (17.18%). Personal hygiene practices were frequently reported. Most participants washed their hands before meals (95.53%), after soil contact (97.25%), and after pet contact (70.45%). In addition, 80.41% reported trimming their nails weekly. Regarding dietary behaviors, 64.60% of participants reported consuming raw vegetables, while 89.69% reported washing vegetables before consumption. Consumption of raw or undercooked meat was reported by 5.15% of participants. Furthermore, 94.16% reported drinking boiled or filtered water.

### 3.4. Factors Associated with Anti-Toxocara spp. IgG Seropositivity

The associations between sociodemographic characteristics, pet management practices, health-related behaviors, and anti-*Toxocara* spp. IgG seropositivity were evaluated using univariate and multivariable logistic regression analyses (Table 4).

Univariate analyses of sociodemographic characteristics showed that higher seroprevalence was observed among residents of Koh Yao Noi, males, participants aged 36–59 years, individuals with an education level of elementary school or below, single participants, unskilled workers, and those with a monthly income of less than USD 300. However, none of these sociodemographic characteristics were significantly associated with *Toxocara* spp. seropositivity (Table 4).

Among the behavioral factors evaluated, only handwashing after cat contact was significantly associated with anti-*Toxocara* spp. IgG seropositivity. Participants who did not wash their hands after cat contact had significantly higher odds of *Toxocara* spp. seropositivity than those who practiced handwashing after cat contact (OR = 1.96; 95% CI: 1.16–3.33; *p* = 0.011) (Table 4). No other behavioral factors were significantly associated with anti-*Toxocara* spp. IgG seropositivity in the univariate analysis. This association remained significant in the multivariable logistic regression model after adjustment for potential confounding factors, with participants who did not practice handwashing after cat contact demonstrating higher odds of *Toxocara* spp. seropositivity (AOR = 1.93; 95% CI: 1.14–3.27; *p* = 0.014) (Table 4).

### 3.5. Comparison of Hematological Parameters Between Seropositive and Seronegative Participants

Hematological parameters were compared between participants who were seropositive and seronegative for anti-*Toxocara* spp. IgG antibodies (Table 5). Seropositive participants had significantly higher mean eosinophil percentages than seronegative participants (5.04 ± 4.14% vs. 3.84 ± 3.43%, *p* = 0.003). A modest but statistically significant difference was also observed in the mean basophil percentage (0.36 ± 0.22% vs. 0.32 ± 0.21%, *p* = 0.048). No significant differences were observed in the remaining hematological parameters (all *p* > 0.05).

## 4. Discussion

*Toxocara* spp. are zoonotic nematodes commonly infecting dogs and cats worldwide and represent an important public health concern as the causative agents of human toxocariasis [2,10]. The high seroprevalence observed in the present study (34.36%) indicates substantial exposure to *Toxocara* spp. among cat owners in Koh Yao District, southern Thailand. This prevalence was lower than that previously reported among primary schoolchildren in Nakhon Si Thammarat Province, Thailand, in 2020 (58.2%) [20] and among dog owners in the same province in 2022 (76.5%) [31], and in several highly endemic settings, including Ho Chi Minh City, Vietnam (45.2%) [21], southern Brazil (64.60%) [36], and Los Baños, Laguna, Philippines (49.0%) [22]. However, it was substantially higher than estimates reported from many developed countries, such as the United States (5.1%) [16], Italy (8%) [18], and Greece (16.0%) [19], as well as from some Asian countries, including Vietnam (13.80%) [37] and China (12.10%) [38]. These findings are consistent with the global epidemiological pattern of *Toxocara* spp. exposure, whereby higher seroprevalence is generally observed in tropical and resource-limited settings characterized by favorable environmental conditions for egg survival, frequent exposure to companion animals, and inadequate environmental sanitation [2,10]. The persistence of environmental exposure to *Toxocara* spp. in such settings may be influenced by the close interactions among humans, companion animals, and contaminated environments, highlighting the importance of a One Health approach to surveillance and prevention.

Seropositivity was highest among residents of Koh Yao Noi (44.33%), followed by Koh Yao Yai (36.11%) and Phru Nai (27.85%), although these differences were not statistically significant. The absence of significant geographical differences suggests relatively widespread exposure to *Toxocara* spp. across the district. Nevertheless, the observed variation in seroprevalence across subdistricts may indicate differences in local environmental and behavioral factors that were not directly assessed in the present study. Previous studies have shown that environmental contamination, density of free-roaming animals, climatic conditions, and hygiene-related behaviors can influence the spatial distribution of anti-*Toxocara* spp. seropositivity [2,10,39,40]. In addition, a previous environmental survey conducted in Koh Yao District detected *Toxocara* spp. eggs in school soils, suggesting the presence of environmental reservoirs that may contribute to human exposure within the community [35].

No significant associations were observed between sociodemographic characteristics and anti-*Toxocara* spp. IgG seropositivity in the present study. Although previous studies have reported inconsistent associations between sociodemographic factors and toxocariasis, behavioral and environmental factors are generally considered to play a more important role in determining exposure [10,40].

The behavioral profile of participants indicated frequent human–cat interactions but limited implementation of preventive cat health care practices. Although many participants reported playing with, touching, kissing, or sleeping with their cats, only a small proportion reported routine deworming, vaccination, or flea and tick control. Previous studies have demonstrated that domestic and free-roaming cats serve as important reservoirs of *Toxocara* spp. and contribute to environmental contamination through the shedding of eggs in feces [8,41]. Consequently, inadequate preventive veterinary care may facilitate environmental contamination and increase opportunities for human exposure. Despite frequent contact with cats, most participants reported good personal hygiene practices, including handwashing before meals, after soil contact, and after defecation, as well as washing vegetables before consumption. These behaviors are recognized as important preventive measures against the accidental ingestion of infective *Toxocara* eggs and other environmentally transmitted parasites [2,40]. The absence of significant associations for behaviors such as playing with, touching, kissing, or sleeping with cats should be interpreted with caution. These behaviors were assessed using binary (yes/no) questions and therefore may not have adequately captured the frequency, duration, or intensity of contact, which are likely to be more relevant indicators of cumulative exposure. In addition, self-reported behavioral data may have been influenced by recall bias and social desirability bias, potentially leading to exposure misclassification. The generally high level of hygiene observed among participants may have reduced the impact of individual behavioral risk factors and may partly explain the lack of significant associations between several behaviors and anti-*Toxocara* spp. IgG seropositivity in the present study.

Regarding modifiable behavioral factors, handwashing after cat contact was the only factor significantly associated with anti-*Toxocara* spp. IgG seropositivity in both the univariate and multivariable analyses. Participants who reported not washing their hands after cat contact had higher odds of anti-*Toxocara* IgG seropositivity than those who did. A similar association was reported among dog and cat owners in Iran, where inadequate handwashing after contact with pets was significantly associated with anti-*Toxocara* IgG seropositivity [29]. Moreover, a study among pet owners in southern Thailand reported that failure to wash hands after animal contact was associated with increased *Toxocara* seropositivity in univariate analysis, highlighting the importance of hand hygiene in reducing exposure to infective eggs [31]. In addition, a recent systematic review and meta-analysis reported that dog and cat contact were significantly associated with human toxocariasis, supporting the importance of pet-associated exposure in disease transmission [28]. However, because the ELISA used in this study detects genus-level anti-*Toxocara* IgG antibodies, it cannot distinguish between *T. cati* and *T. canis*. Although *Toxascaris leonina* can infect both cats and dogs, the commercial ELISA used in this study is designed to detect antibodies against *Toxocara* spp. and does not specifically assess serological exposure to *T. leonina*. Furthermore, although the study population was drawn from a predominantly Muslim island community, where dog ownership is generally uncommon because of cultural and religious practices, contact with free-roaming dogs may still occur. Therefore, exposure to *T. canis* cannot be excluded, and the observed seropositivity should be interpreted as evidence of *Toxocara* spp. exposure among cat owners rather than exposure attributable specifically to cats. However, *Toxocara* eggs shed in feline feces are not infective immediately after excretion and require at least two weeks in the environment to embryonate and reach the infective stage [11]. Therefore, direct transmission from a cat’s fur is likely to be less important than indirect exposure through contaminated soil, litter boxes, or other shared environmental surfaces. Consequently, the observed association between handwashing after cat contact and lower anti-*Toxocara* spp. IgG seropositivity may reflect reduced contact with contaminated environmental sources rather than direct removal of infective eggs acquired from cat fur. Previous studies have also reported an association between inadequate hand hygiene and human toxocariasis and other environmentally transmitted helminth infections [2,40]. Interestingly, other cat-related behaviors, including playing with, touching, kissing, or sleeping with cats, were not significantly associated with anti-*Toxocara* spp. IgG seropositivity. Although pet ownership and animal contact have been associated with an increased risk of toxocariasis [28], the specific cat-related behaviors evaluated in the present study may not adequately reflect cumulative exposure to embryonated *Toxocara* eggs. Humans primarily acquire toxocariasis through the accidental ingestion of embryonated eggs present in contaminated soil, food, water, or other environmental sources [2]. Previous environmental investigations conducted in Koh Yao District detected *Toxocara* spp. eggs in school soils, particularly in rural areas, indicating the presence of environmental reservoirs within the community [35]. These findings suggest that environmental contamination may contribute to *Toxocara* spp. exposure in addition to direct or indirect contact with cats. However, because environmental samples were not collected concurrently with the present study, this potential relationship should be interpreted with caution. Although good hand hygiene may reduce opportunities for exposure to embryonated *Toxocara* eggs present in contaminated environmental sources, the cross-sectional design does not allow temporal or causal inference. Nevertheless, promoting appropriate hand hygiene remains a simple and practical public health measure that may help reduce opportunities for *Toxocara* spp. exposure, particularly among pet owners and individuals living in endemic communities. Collectively, these findings suggest that exposure to *Toxocara* spp. may occur through multiple transmission pathways and highlight the need for integrated prevention strategies, including hygiene promotion, environmental sanitation, and responsible pet ownership.

Hematological analysis revealed significantly higher eosinophil percentages among seropositive individuals. Similar findings have been reported in previous studies of human toxocariasis and other helminth infections, where eosinophilia was frequently observed among infected individuals [31,42,43]. Elevated eosinophil levels are a well-recognized immunological response to helminth infections and have been associated with larval migration and tissue invasion in toxocariasis [40]. Eosinophils contribute to host defense through the release of cytotoxic granules, reactive oxygen species, and inflammatory mediators that participate in parasite killing and modulation of immune responses [42]. Although anti-*Toxocara* IgG seropositivity reflects previous exposure rather than active infection, the higher eosinophil percentages observed among seropositive participants are consistent with an immune response associated with previous *Toxocara* spp. exposure. However, this finding should be interpreted with caution because eosinophilia is a nonspecific response and may occur in a variety of parasitic infections and allergic conditions.

A modest but statistically significant increase in basophil percentages was also observed among seropositive participants. Although basophils are involved in type 2 immune responses associated with helminth infections [44,45], the biological significance of this finding remains uncertain because the difference between groups was small, basophil counts remained within the normal reference range, and no significant differences were observed in the other hematological parameters.

This study has several limitations. First, the sample size calculation was based on the reported seroprevalence among dog owners rather than cat owners due to the lack of published data on *Toxocara* seroprevalence among cat owners in Thailand at the time of study design. Although this represented the most geographically relevant evidence available, differences in pet ownership, environmental contamination, and ecological characteristics between the study populations may have influenced the underlying prevalence of *Toxocara* exposure. Second, the cross-sectional design precludes the establishment of causal relationships between the identified factors and anti-*Toxocara* spp. IgG seropositivity. In addition, participants were recruited using a convenience sampling approach, and the predominance of female participants may reflect the availability and willingness of household members to participate during data collection rather than the actual demographic distribution of cat owners in the community. Therefore, the study population may not be fully representative of all cat owners in Koh Yao District. Third, the detection of anti-*Toxocara* IgG antibodies by ELISA indicates exposure to *Toxocara* spp. but cannot distinguish between previous exposure and current infection or differentiate between *T. cati* and *T. canis*. Furthermore, information on dog ownership and dog contact was not collected. Therefore, the species responsible for the observed seropositivity could not be determined. Future studies incorporating molecular and parasitological methods, together with data on dog ownership, dog contact, and environmental contamination, may provide a more comprehensive understanding of the sources of *Toxocara* exposure and active infection. Fourth, although ELISA is widely used in seroepidemiological investigations, it is not considered a definitive diagnostic test for human toxocariasis. Cross-reactivity with antibodies against other helminths, particularly Ascaris lumbricoides, may result in false-positive reactions [46,47]. Nevertheless, previous studies conducted in southern Thailand, including Koh Yao District, have not reported *Ascaris lumbricoides* infection. Instead, hookworm and *Trichuris trichiura* were detected at low prevalence, ranging from 1.24% to 4.11% for hookworm and 1.24% to 1.65% for *T. trichiura* [33,48]. Although TES-based ELISA may exhibit cross-reactivity with antibodies against other helminths, clinically significant cross-reactivity has been reported predominantly with *A. lumbricoides*. Evidence for cross-reactivity with hookworm or *T. trichiura* is limited. Therefore, although cross-reactivity cannot be completely excluded, it is unlikely to have substantially influenced the seroprevalence estimates in the present study. Finally, environmental samples and fecal samples from cats owned by the study participants were not collected. Therefore, potential environmental sources of *Toxocara* spp. exposure, the infection status of household cats, and their relationship with anti-*Toxocara* spp. IgG seropositivity among cat owners could not be directly assessed. Further studies integrating human, animal, and environmental investigations within a One Health framework are warranted to better understand the epidemiology and transmission dynamics of *Toxocara* spp. in island communities.

## 5. Conclusions

This study demonstrated a high seroprevalence of anti-*Toxocara* spp. IgG antibodies among cat owners in Koh Yao District, Phang Nga Province, Thailand, indicating substantial exposure to *Toxocara* spp. within the community. Handwashing after cat contact was independently associated with lower odds of anti-*Toxocara* spp. IgG seropositivity, highlighting the potential importance of personal hygiene in reducing opportunities for exposure to embryonated *Toxocara* eggs in contaminated environments. In addition, seropositive individuals exhibited significantly higher eosinophil percentages than seronegative individuals, although this finding should be interpreted cautiously because anti-*Toxocara* spp. IgG seropositivity reflects previous exposure rather than active infection. These findings underscore the need for targeted health education programs promoting hand hygiene and responsible pet care among cat owners and other at-risk populations. Further environmental investigations and longitudinal studies within a One Health framework are warranted to improve understanding of transmission dynamics and support the development of effective prevention and control strategies in endemic communities.

## Figures and Tables

**Table 1:** Sociodemographic characteristics (*n* = 291).

Characteristics	Number	Percentage
Subdistrict		
Koh Yao Yai	36	12.37
Phru Nai	158	54.30
Koh Yao Noi	97	33.33
Sex		
Male	41	14.09
Female	250	85.91
Age group (years)		
18–35	61	20.96
36–59	155	53.26
≥60	75	25.77
Education		
≤Elementary school	154	52.92
>Elementary school	137	47.08
Religion		
Buddhist	5	1.72
Islam	286	98.28
Marital status		
Single	59	20.27
Married	217	74.57
Widowed/divorced	15	5.15
Occupation		
Unskilled workers	267	91.75
Skilled workers	24	8.25
Income		
Less than USD 300	119	40.89
USD 300 or more	172	59.11

**Table 2:** Seroprevalence of anti-*Toxocara* spp. IgG antibodies among cat owners in Koh Yao District, Phang Nga Province, Thailand (*n* = 291).

Characteristics	Total, *n* (%)	Seropositivity, *n* (%)
Subdistrict		
Koh Yao Yai	36 (12.37)	13 (36.11)
Phru Nai	158 (54.30)	44 (27.85)
Koh Yao Noi	97 (33.33)	43 (44.33)
Sex		
Male	41 (14.09)	15 (36.59)
Female	250 (85.91)	85 (34.00)
Age group (years)		
18–35	61 (20.96)	20 (32.79)
36–59	155 (53.26)	54 (34.84)
≥60	75 (25.77)	26 (34.67)
Education		
≤Elementary school	154 (52.92)	60 (38.96)
>Elementary school	137 (47.08)	40 (29.20)
Marital status		
Single	59 (20.27)	22 (37.29)
Married	217 (74.57)	74 (34.10)
Widowed/divorced	15 (5.15)	4 (26.67)
Occupation		
Unskilled workers	267 (91.75)	94 (35.21)
Skilled workers	24 (8.25)	6 (25.00)
Income		
Less than USD 300	119 (40.89)	43 (36.13)
USD 300 or more	172 (59.11)	57 (33.14)
Overall	291 (100.00)	100 (34.36)

**Table 3:** Health-related behaviors of study participants in Koh Yao District, Phang Nga Province, Thailand (*n* = 291).

Behaviors	Practice, *n* (%)	
	No	Yes
Cat deworming (every 3 months)	278 (95.53)	13 (4.47)
Cat bathing/grooming (weekly)	262 (90.03)	29 (9.97)
Flea/tick control for cats	269 (92.44)	22 (7.56)
Playing with cats	174 (59.79)	117 (40.21)
Kissing cats	223 (76.63)	68 (23.37)
Touching cats	182 (62.54)	109 (37.46)
Sleeping with cats	241 (82.82)	50 (17.18)
Handwashing before meals	13 (4.47)	278 (95.53)
Handwashing after soil contact	8 (2.75)	283 (97.25)
Handwashing after pet contact	86 (29.55)	205 (70.45)
Nail trimming (weekly)	57 (19.59)	234 (80.41)
Raw vegetable consumption	103 (35.40)	188 (64.60)
Washing vegetables before consumption	30 (10.31)	261 (89.69)
Raw/undercooked meat consumption	276 (94.85)	15 (5.15)
Drinking boiled/filtered water	17 (5.84)	274 (94.16)

**Table 4:** Factors associated with anti-*Toxocara* spp. IgG seropositivity among cat owners in Koh Yao District, Phang Nga Province, Thailand.

Variables	N ^1^	Seropositive ^2^	OR ^3^ (95% CI)	*p*-Value	AOR ^4^ (95% CI)	*p*-Value
Subdistrict						
Koh Yao Yai	36 (12.37)	13 (36.11)	Ref.			
Phru Nai	158 (54.30)	44 (27.85)	0.68 (0.31–1.47)	0.328		
Koh Yao Noi	97 (33.33)	43 (44.33)	1.41 (0.64–3.10)	0.395		
Sex						
Male	41 (14.09)	15 (36.59)	Ref.			
Female	250 (85.91)	85 (34.00)	0.89 (0.44–1.76)	0.747		
Age group (years)						
18–35	61 (20.96)	20 (32.79)	Ref.			
36–59	155 (53.26)	54 (34.84)	1.10 (0.58–2.05)	0.775		
≥60	75 (25.77)	26 (34.67)	1.09 (0.53–2.22)	0.818		
Education						
≤Elementary school	154 (52.92)	60 (38.96)	Ref.		Ref.	
>Elementary school	137 (47.08)	40 (29.20)	0.65 (0.40–1.06)	0.081	0.66 (0.40–1.09)	0.104
Marital status						
Single	59 (20.27)	22 (37.29)	Ref.			
Married	217 (74.57)	74 (34.10)	0.87 (0.48–1.58)	0.649		
Widowed/divorced	15 (5.15)	4 (26.67)	0.61 (0.17–2.16)	0.444		
Occupation						
Unskilled workers	267 (91.75)	94 (35.21)	Ref.			
Skilled workers	24 (8.25)	6 (25.00)	0.61 (0.24–1.60)	0.317		
Income						
Less than USD 300	119 (40.89)	43 (36.13)	Ref.			
USD 300 or more	172 (59.11)	57 (33.14)	0.88 (0.54–1.43)	0.597		
Cat deworming (every 3 months)						
Yes	13 (4.47)	2 (15.38)	Ref.		Ref.	
No	278 (95.53)	98 (35.25)	3.03 (0.65–14.26)	0.159	2.92 (0.62–13.75)	0.176
Cat bathing (weekly)						
Yes	29 (9.97)	10 (34.48)	Ref.			
No	262 (90.03)	90 (34.35)	0.99 (0.44–2.22)	0.989		
Flea/tick control for cats						
Yes	22 (7.56)	5 (22.73)	Ref.			
No	269 (92.44)	95 (35.32)	1.85 (0.66–5.26)	0.238		
Playing with cats						
No	174 (59.79)	62 (35.63)	Ref.			
Yes	117 (40.21)	38 (32.48)	0.87 (0.53–1.43)	0.579		
Kissing cats						
No	223 (76.63)	79 (35.43)	Ref.			
Yes	68 (23.37)	21 (30.88)	0.81 (0.45–1.46)	0.490		
Touching cats						
No	182 (62.54)	65 (35.71)	Ref.			
Yes	109 (37.46)	35 (32.11)	0.85 (0.51–1.41)	0.531		
Sleeping with cats						
No	241 (82.82)	82 (34.02)	Ref.			
Yes	50 (17.18)	18 (36.00)	1.09 (0.58–2.06)	0.789		
Handwashing before meals						
Yes	278 (95.53)	95 (34.17)	Ref.			
No	13 (4.47)	5 (38.46)	1.21 (0.38–3.87)	0.751		
Handwashing after soil contact						
Yes	283 (97.25)	98 (34.63)	Ref.			
No	8 (2.75)	2 (25.00)	0.63 (0.12–3.25)	0.575		
Handwashing after cat contact						
Yes	205 (70.45)	61 (29.76)	Ref.		Ref.	
No	86 (29.55)	39 (45.35)	1.96 (1.16–3.33)	0.011	1.93 (1.14–3.27)	0.014
Nail trimming (Weekly)						
Yes	234 (80.41)	80 (34.19)	Ref.			
No	57 (19.59)	20 (35.09)	1.04 (0.57–1.92)	0.898		
Raw vegetable consumption						
No	103 (35.40)	34 (33.01)	Ref.			
Yes	188 (64.60)	66 (35.11)	1.10 (0.66–1.83)	0.719		
Washing vegetables before consumption						
Yes	261 (89.69)	88 (33.72)	Ref.			
No	30 (10.31)	12 (40.00)	1.32 (0.61–2.86)	0.493		
Raw/undercooked meat consumption						
No	276 (94.85)	94 (34.06)	Ref.			
Yes	15 (5.15)	6 (40.00)	1.29 (0.45–3.74)	0.638		
Drinking boiled/filtered water						
Yes	274 (94.16)	91 (33.21)	Ref.		Ref.	
No	17 (5.84)	9 (52.94)	2.27 (0.85–5.88)	0.104	2.14 (0.79–5.82)	0.135

^1^ Total sample of 291; ^2^ Total anti-*Toxocara* spp. IgG seropositive participants = 100; ^3^ Odds ratio (OR); ^4^ Adjusted odds ratio (AOR).

**Table 5:** Comparison of hematological parameters between anti-*Toxocara* spp. IgG seropositive and seronegative participants.

Blood Parameters	Normal Ranges	Groups		*p*-Value
		Seropositive (*n* = 100)	Seronegative (*n* = 191)	
RBC (×10^6^/μL)	3.80–5.80	4.37 ± 0.48	4.33 ± 0.48	0.371
Hemoglobin (g/dL)	11.00–16.50	11.73 ± 1.26	11.61 ± 1.26	0.137
Hematocrit (%)	35.00–50.00	36.36 ± 3.67	36.14 ± 3.85	0.179
MCV (fL)	80.00–98.00	83.69 ± 8.57	83.85 ± 8.01	0.882
MCH (pg)	27.00–32.00	26.96 ± 2.72	26.91 ± 2.52	0.769
MCHC (g/dL)	32.00–36.00	32.27 ± 1.55	32.13 ± 1.50	0.496
RDW-SD (fL)	37.00–54.00	41.93 ± 3.65	42.27 ± 4.27	0.736
RDW-CV (%)	11.50–14.50	14.61 ± 2.80	15.02 ± 7.47	0.999
PDW (fL)	9.00–17.00	11.32 ± 2.06	11.14 ± 1.59	0.875
MPV (fL)	7.50–12.00	10.14 ± 1.42	10.11 ± 1.21	0.943
P-LCR (%)	13.00–43.00	25.68 ± 7.65	25.35 ± 6.16	0.942
WBC (×10^3^/μL)	5.00–10.00	6.92 ± 1.55	6.87 ± 1.67	0.507
Neutrophils (%)	40.00–80.00	51.57 ± 8.96	53.65 ± 9.15	0.065
Lymphocytes (%)	20.00–70.00	37.51 ± 7.51	36.74 ± 8.68	0.456
Monocytes (%)	2.00–10.00	5.62 ± 1.61	5.50 ± 1.41	0.776
Eosinophils (%)	0.00–6.00	5.04 ± 4.14	3.84 ± 3.43	0.003 *
Basophils (%)	0.00–2.00	0.36 ± 0.22	0.32 ± 0.21	0.048 *
Platelet (×10^3^/μL)	140.00–400.00	304.75 ± 90.74	294.69 ± 77.35	0.226

Note: 1. All hematological parameters, except neutrophil (%) and lymphocyte (%), were compared using the Mann–Whitney U test. 2. Neutrophil (%) and lymphocyte (%) were compared using the independent-samples *t*-test. 3. * Statistically significant at *p*-value < 0.05.

## Data Availability

The data supporting the findings of this manuscript are available from the corresponding authors upon reasonable request.
